# Awareness of Dysphagia-Related Complications and Risks and the Importance of Early Intervention in Patients with Parkinson's Disease: A Qualitative Study

**DOI:** 10.1155/2023/9514851

**Published:** 2023-03-03

**Authors:** Kaifeng Yao, Lihua Wang, Lihua Zhang

**Affiliations:** ^1^Department of Neurology, Affiliated Hospital No. 2 of Nantong University, Nantong 226000, China; ^2^Department of Nursing, Affiliated Hospital No. 2 of Nantong University, Nantong 226000, China

## Abstract

**Objective:**

To investigate the awareness of dysphagia-related complications and risks and the importance of early intervention in patients with Parkinson's disease (PD).

**Methods:**

Using the phenomenological approach of the qualitative study, 18 patients with PD in a Grade A tertiary hospital in Nantong were selected, and semistructured personal in-depth interviews were conducted. The interview content was analyzed using Colaizzi's seven-step method, and the topics and subtopics were further refined.

**Results:**

Awareness of dysphagia-related complications and risks and the importance of early intervention in patients with PD can be summarized into three topics: lack of knowledge about PD and dysphagia, changes in emotional cognition, and low need for early intervention for dysphagia.

**Conclusions:**

Patients with PD have a low awareness of dysphagia, do not follow any preventative measures, and have difficulty in recognizing the disease symptoms; hence, there is a vital need for early intervention. Medical staff need to create awareness among patients and their families, provide health education through multiple channels, popularize the knowledge of PD complications such as dysphagia, improve patient compliance with respect to medication, regular consultation, and medical treatment, guide the transformation of negative emotions in patients to positive emotions, and help patients with PD to actively prevent dysphagia and other complications and improve their quality of life.

## 1. Introduction

Parkinson's disease (PD) is the second most prevalent neurodegenerative disease among the elderly after Alzheimer's disease, [1] affecting 3% of the global population [2]. It is caused by a decrease in the neurotransmitter dopamine level and associated nigrostriatal neurodegeneration [3]. It has both motor symptoms, such as static tremor, myotonia, and slow movement and nonmotor symptoms, such as autonomic dysfunction and mental symptoms [4, 5].

One of the most common nonmotor symptoms of PD, oropharyngeal dysphagia is mostly caused by gastrointestinal dysfunction and autonomic nerve disorder. As its clinical significance has become increasingly apparent in recent years, dysphagia has steadily attracted the attention of researchers. The prevalence of dysphagia in PD ranges between 18.5% and 100%, a variation that can be explained by the use of different methodologies for assessing swallowing function or the level of PD [6].

Aspiration pneumonia, a complication of oropharyngeal dysphagia (OPD), is the leading cause of mortality in PD. The symptoms of OPD can manifest in the early stages of PD, and 40–78% of patients exhibit changes in swallowing function [7]. In the early stages of PD, medical personnel frequently overlook symptoms that respond to dysphagia. Some studies have found that 50% of patients with PD may have unperceived dysphagia in the early stages, and occult aspiration accounts for 15% of such cases. As per the results of some studies, dysphagia in patients with PD may lead to a small amount of drug residue in the pharynx [8, 9]. The impact of the medication is then solely affected by residue or tablet adaptation, such as when the tablets are crushed. In the late stage of PD with severe dysphagia, patients need to be intubated [6]. According to a study, the average survival time after OPD onset is only two years [7, 10]. Therefore, dysphagia is one of the most serious complications in patients with PD.

In addition to causing various oropharyngeal symptoms in patients with PD, such as excessive saliva, patients suffering from PD could have excessive production of saliva or reduction in the ability to swallow (reduced number of swallows per minute) saliva in the mouth and to cough, aspiration pneumonia, and asphyxia. Dysphagia can also lead to the occurrence or even aggravation of various negative emotions, such as anxiety, depression, shame, loneliness, inferiority complex, and self-image disorder, thereby affecting patients' communication and social interaction. There are several studies in China that focus on these factors and related negative emotions [11–14]. In 2020, the fourth edition of the China Parkinson's Disease Treatment Guidelines highlighted in the chapter on rehabilitation exercise therapy that medication is less effective in treating nonmotor symptoms such as dysphagia; instead, rehabilitation and exercise therapy can be of assistance.

In 2021, a consensus group, with Italian neurologists as the core, formed a multinational consensus on the screening, diagnosis, and prognostic value of dysphagia in PD [15]. Researchers have conducted multiple studies on PD with dysphagia, but there are few qualitative studies on the cognitive aspects of the complications in patients with PD with dysphagia. Considering that dysphagia has a definitive impact on the prognosis of patients with PD, in this study, we conducted in-depth interviews with patients with PD who did not have obvious dysphagia symptoms to comprehend their awareness of dysphagia and related intervention, to provide a theoretical basis for developing early personalized intervention programs for patients with PD.

## 2. Participants and Methods

### 2.1. Participants

Using the purposeful sampling method, patients with PD in a Grade A tertiary hospital in Nantong were selected as the interview respondents.

The inclusion criteria were as follows: (1) diagnosis of primary PD was in line with the 2015 movement disorder society's clinical diagnostic criteria for Parkinson's disease, (2) patients were aged ≤80 years old, (3) patients were aware of their own disease diagnosis and condition, and (4) patients were informed of and agreed to be interviewed.

The exclusion criteria were as follows: (1) patients without PD; (2) patients with cognitive impairment and communication impairment; (3) patients with other serious diseases that may cause dysphagia, such as stroke and esophageal cancer; and (4) patients with a history of psychological disorders or mental illness.

The sample size was supported by the saturated interview data; that is, no new themes emerged from the interview content of the interviewees. A total of 18 patients, numbered N1–N18, were interviewed. This study, numbered 2020KT171, was approved by the hospital's ethics committee.

### 2.2. Methods

#### 2.2.1. Data Collection Method

*(1) Study Design*. Using the phenomenological approach [16] of the qualitative study, we conducted one-on-one semistructured interviews to collect data. Based on the purpose of the study and referring to literature, after consultation with two authoritative PD therapists on neurology and preinterviews with two patients with PD, the outline of this study was drawn up with the following interview questions:

1. As per your knowledge, what are the early symptoms of PD?
2. Do you know how PD affects swallowing?
3. As per your knowledge, what are the symptoms of deglutition disorders in PD?
4. Is there any difference in your current eating and drinking habits compared to previously? If yes, then please specify
5. At times, do you drool?
6. Are these manifestations bothering you?
7. Do you know what will happen if a deglutition disorder worsens?
8. What do you do at home if you have drooling, or you are unable to swallow?
9. Do you speak to your relatives or medical staff about your symptoms?
10. Do you think the support and concern of your loved ones are important?
11. What kind of help do you hope to get for the deglutition disorder caused by PD?
12. Are you willing to receive related exercise therapy to prevent PD deglutition disorders, such as swallowing training, voice training, and singing therapy?

*(2) Data Collection*. The researchers conducted in-depth interviews with 18 patients with PD according to the interview outline. Before data collection, the researchers introduced themselves to the interviewees and explained the significance, purpose, and main content of this study; the interviewees then signed the informed consent form. The interview times ranged from 28 to 56 minutes. During the interviews, which were recorded, the researchers listened attentively, focused on the patients' facial expressions, movements, and emotional reactions, did not interrupt them, and did not ask induced questions. All interviews were completed by the researchers themselves.

*(3) Data Analysis*. Data collection and analysis were conducted concurrently. At the end of each interview, the recorded audio was transcribed within 24 hours, and the interview data were analyzed. The phenomenological Colaizzi's method [17] was used for a seven-step analysis. The data were transcribed, coded, and analyzed by two people, and then refined, and finally defined as a reasonable and logical topic and subtopic.

#### 2.2.2. Quality Control

Researchers involved in this study were engaged in clinical nursing in the Department of Neurology for 18 years. Nursing more than 80 patients with PD every year, they have accumulated rich theoretical understanding and practical experience in the treatment and nursing of patients with PD. Prior to conducting the interviews, the researchers participated in several online training courses on scientific research methods, such as qualitative study, organized by the Chinese Nursing Association. A nursing expert with rich experience in qualitative research and who has experience supervising graduate students in qualitative research made five revisions to the first draft of the interview outline developed by the research team. The nursing expert then instructed the researchers on how to properly select research participants, how to choose qualitative research methods such as rooting theory, ethnography, narrative research, and phenomenology, and on informing the reasons for choosing the selected method. Then, based on the preexperimental interview recordings, the researchers were instructed on the proper use of interview techniques to obtain more realistic and comprehensive information about the patients.

First, the researchers selected different representative interviewees according to their age, occupation, gender, and educational background. At the beginning of the interview, they informed the interviewees about the purpose, significance, content of this study, and the principles of voluntariness and confidentiality. After obtaining the informed consent, they recorded each interview onsite and ensured the privacy of the patients was protected. During the interview process, they used communication skills to gain the trust of the patients and asked open-ended questions according to the outline. The interview site was the No. 21 consulting room next to the neurology expert clinic, the environment was peaceful, with sufficient illumination, and the interview was not likely to be disturbed. This ensured that the interviewees could express their true feelings and thoughts without reservation.

## 3. Results

### 3.1. Basic Information

The basic information of the 18 interviewees such as gender, age, occupation, education level, payment of medical expenses, course of disease, and type of medication is listed in Table 1. The different ages, occupations, and course of disease could ensure that the respondents were representative to saturate the sample.

### 3.2. Topic Results (Table 2)

The topic results are shown in Table 2 as follows:

#### 3.2.1. Topic 1: Lack of Knowledge about PD and Dysphagia

*(1) Insufficient Awareness of Common Symptoms of PD*. Common symptoms of PD include motor and nonmotor symptoms. Motor symptoms include static tremor, myotonia, bradykinesia, and postural balance disorders. Nonmotor symptoms include smell disorder, sleep disorder, autonomic dysfunction (constipation, dysphagia, and excessive salivation), and cognitive and mental disorders [18]. When the interviewees were asked what the symptoms of PD were, most of the patients only spoke of their own symptoms and knew nothing about others. Also, the answers of 16/18 of the respondents focused on common symptoms, such as static tremor. The results of this study showed that patients with PD had insufficient awareness of disease symptoms, and after being informed and educated about the disease, they expressed surprise that PD could affect swallowing and lead to dysphagia.

  N1: “I only know that this disease will lead to tremors in the hand or foot. I have tremors in my right hand, while my left hand functions well. I also have constipation. I have had good bowel movements recently, and the condition is much better than that a few days ago. I have not heard that it affects swallowing, and I havenot experienced it yet. I have a good appetite.”
  N2: “There are few people who suffer from this disease. Sometimes, my left hand trembles when I am nervous, and I react slowly. I am a weighing clerk in the supermarket. Sometimes, I tremble badly when I am busy.”
  N4: “After I got this disease, my reactions have slowed down. When sitting in a chair, I feel like a tendon in my leg is being stretched, but I feel okay when I stand up and walk. I do not know what the other symptoms are. No one has told me about them. Every time I see a doctor, I receive advice on my medication.”
  N9: “I have tremors in my hands and feet, due to which it is difficult to walk. That is the only problem. I do not talk as fluently as before. I usually chant sutras to others. I find that tremors in my hands and feet are not related to me being nervous. During my re-examination, I was instructed to change the dosage of Madopar to half a capsule, but the effect was not obvious. I do not know about other symptoms.”
  N11: “I just feel uncomfortable in my hands and feet, I cannot sleep well at night, and I often have nightmares. I do not know about other symptoms.”
  N17: “I like to read books. I have also checked about the disease on the internet. I know that this disease involves many symptoms, such as tremors in the hands and feet, muscle stiffness, and difficulty in walking. It is said that it can be treated with surgery, isn't that so?”

*(2) No Awareness of Excessive Salivation, the Main Symptoms of Oropharyngeal Dysphagia*. Patients with PD may have dysphagia in the early stage, and the clinical manifestations vary. In addition to the main complaint of OPD, it can also manifest as excessive salivation, food residue in the oral cavity after eating, a slow-down in oropharyngeal swallowing, stagnation of bolus, and aspiration [19]. However, 80% of patients with excessive salivation symptoms in this study were not aware that excessive salivation was also a manifestation of OPD. There were several situations:

1. The doctor did not inform the patient. N4: “I sometimes produce a lot of saliva, especially when I sleep. Is too much saliva also a symptom of OPD? I never mentioned it to the doctor.”
2. Symptoms were ignored. N16: “Sometimes, when I sleep, I produce a lot of saliva. My wife dislikes it that my pillow is wet with saliva. The situation is good during the day. I have no trouble eating. I do not choke, but I just eat slowly.” N8: “I do not choke when I eat or drink. I have no problem swallowing.”
3. Concerned about personal image. N12: “During the day, sometimes when I talk, I feel like I am going to drool, which makes me embarrassed to talk to others. I did not know that it is also a symptom of my disease. I was afraid of being laughed at, so I did not speak about it previously.”

*(3) Danger of Oropharyngeal Dysphagia Is Unclear*. Dysphagia in patients with PD can lead to various complications, such as insufficient drug intake, malnutrition, dehydration, or secondary pneumonia, which is the leading cause of death in patients with PD [20]. The results of this study showed that most of the respondents were unaware of the consequences of OPD and even undervalued or underestimated them after being informed.

  N2: “I did not know, I am fine now anyway”
  N5: “I can cough. Sometimes, I feel discomfort in my throat when I swallow something. I have to lift my neck to make it easier to swallow. I am also worried that it will worsen. Is there any serious consequence?”
  N9: “Compared with previously, there are indeed some changes in swallowing. When I take medicine, even though I swallow water several times, the tablets remain in my mouth. It seems that swallowing has slowed down.”

*(4) Lack of Coping Measures for Symptoms of Dysphagia*. The results of this study showed that most patients do not know how to suitably face and take effective preventive measures in the early stage of dysphagia, such as salivation or slow swallowing; also, the symptoms of most patients are accompanied by “on-off phenomenon” due to medication effect time, [21] due to which the patient does not know when they will occur; this is related to the medication and health education. The main reasons for not knowing the intervention measures are as follows:

1. There was no relevant guidance at the time of consulting a doctor. N14: “Drooling is much better now. I drool a lot when I sleep. Sometimes my pillow is wet, but I cannot help it. The doctor just tells me to take medicine.” N3: “In such a case, I only take medicine. The outpatient doctor did not tell me how to deal with this.”
2. Access to information. N11: “I drool, sometimes it is light and sometimes it is severe, and my voice has become lower. I do not know how to prevent this situation from getting worse, but I only take medicine. I use the mobile phone designed for elderly people and I cannot read.”
3. Lack of awareness of reexamination and self-medication. N16: “I have been buying medicine from pharmacies all the time. Last time, I changed my Madopar dose from a quarter to a half tablet.”
4. Expressing of comprehension. N17: “The doctor has not mentioned this. The outpatient doctor is busy and has no time to talk to us about it, but I think we should pay attention to our own disease and actively learn relevant knowledge.”

#### 3.2.2. Topic 2: Emotional Cognitive Changes

*(1) Anxiety and Depression*. About 35% of patients with PD have depression, and 31% of patients with PD have anxiety, among whom, patients with both depression and anxiety account for the majority [22, 23]. In the context of COVID-19, due to the difficulty in consulting with doctors, patients with PD were more stressed, and their anxiety and depression levels were worse [24]. Most of the interviewed patients were worried about the inconvenience of visiting a doctor.

  N8: “I feel that my condition is getting worse. I am not interested in anything. I used to play cards to pass the time, but now I cannot hold the cards as my hands are shaking. I do not go out alone because I do not walk very quickly and I am afraid that the villagers will laugh at me.”
  N18: “My son and daughter are very kind to me, but they must go to work. I used to do all the housework at home, but now I feel as though I am useless.”

*(2) Sense of Powerlessness*. Powerlessness is a psychosocial phenomenon. As a nursing diagnosis, powerlessness was first incorporated into North America in 1982. It refers to a perception that a person's behavior will not have a significant impact on the results, and there is a lack of control over the current situation and what will happen.

  N3: “This disease cannot be healed anyway. It cannot be cured, no matter how much medicine I take, and there are also side effects. I think it is too uncomfortable to take amantadine, so I stopped taking it. If complications really happen, there is nothing I can do about it.”

#### 3.2.3. Topic 3: Need for Early Intervention of Dysphagia

The proportion of patients with PD with occult dysphagia, [25] which needs early evaluation and prevention is high [26]. The results of this study showed that patients with PD have different attitudes towards the need for early intervention of dysphagia.

*(1) Interested in Intervention*. Through the interviews, it was found that 65% of the patients had a positive desire for intervention and hoped to get help from medical staff to prevent dysphagia through guidance. Two patients showed keen interest in early intervention of PD complications.

  N8: “I am often unhappy at home. It is great that there is a way for me to prevent complications. If you start the singing therapy course, please inform me about it. I will wait for your update at home.”
  N9: “I must participate in this training. It is good for me.”

*(2) Not Interested in Intervention*. In this study, three respondents maintained an indifferent attitude towards early preventive measures.

  N16: “I am willing to participate in this training for early prevention of complications, but I am busy recently and may not be free to participate in the training. Let us talk about it later.”

*(3) Concerns*. In this study, five patients had various concerns about the early intervention of dysphagia.

1. Considering self-image. N10: “Okay, I am willing to participate, and I will cooperate in the exercise, but I hope to be taught alone because I do not think it is good that people recognize me during the class.” N1: “I used to work in government offices. My symptoms are not remarkable unless I am nervous. I do not want to participate in training, which may let other people know that I have Parkinson's disease. It is not good for me.”
2. Impact of family support system. N11: “My grandson accompanies me to get my medicine prescription during the holidays. Usually, I am at home alone and no one cares about me. I cannot participate in these activities or sing.” N17: “My son asked for a leave of one hour to accompany me to see a doctor. Time is very tight. I have to leave after getting the medication. I do not think it is necessary nor do I have the time to do voice exercise.” N4: “Yes, I still have low blood pressure. My daughter always blames me for thinking too much. It is obviously not a big deal.”

## 4. Discussion

### 4.1. Strengthening Health Education and Correcting Misconceptions

The results of this study showed that outpatients with PD had low or no knowledge of PD symptoms other than motor symptoms and that 16/18 of the respondents did not know or pay attention to one of the most serious complications, swallowing disorder, and only 6/18 of the respondents were willing and wanted early intervention to delay the onset of swallowing disorders, which is in line with the other findings of this study that patients with PD have low knowledge of the disease and its complications. There are few qualitative studies on the knowledge and intervention needs for PD complications among patients [27]; however, studies have pointed out the need for early interventions to delay the onset of dysphagia complications in the early stages of PD.

One of the common complications of PD, dysphagia can lead to various other complications in patients, such as pneumonia, dehydration, and malnutrition. It can even aggravate emotional problems, such as anxiety and depression, and can directly or indirectly affect the quality of life of patients, accelerate the progress of the disease, and have a negative impact on the prognosis [6, 15, 28]. In addition to the burden of the disease itself, dysphagia may also cause negative changes in patients' self-confidence, self-image, and understanding of roles and social functions and damage their social interactions and communication behavior [29]. The National Institute for Health and Care Excellence [30] proposed the diagnosis and management methods for adult patients with PD and required that patients with communication disorders, dysphagia, and excessive saliva should be given speech and language therapy to improve their communication and speech functions and reduce the risk of aspiration. The results of this study show that most patients lack understanding of dysphagia in the early stage of PD. They do not know that excessive salivation or anterior spillage or saliva accumulation is a manifestation of dysphagia and lack coping measures for dysphagia. Patients with PD are mostly outpatients, who cannot rely on medical treatment to get health education and other services due to heavy outpatient work. Hospitals have not set up outpatient nurses for nursing special diseases, so patients cannot get standardized and comprehensive disease-related health education and nursing guidance, and the needs of various specialized assessments cannot be met. The results of this study also revealed that due to misunderstanding the condition, some patients with PD had a low rate of outpatient follow-up, and the longest follow-up time was 10 months. The opportunity to remedy such incorrect understanding and medication behavior in time was missed, resulting in an aggravation of the symptoms.

Xue et al. [31] studied and confirmed that the establishment of specialized care clinics can reduce the rehospitalization rate of patients and improve overall satisfaction. Calabresi et al. [32] confirmed through experiments that although drug and physical therapy can positively affect the clinical manifestations of PD, the guiding nursing management led by PD nurses may be the key to a better quality of life and higher patient compliance. Therefore, it is recommended to set up an intrinsic outpatient facility for PD-specific diseases, where medical guidance is provided by different professionals, specialist nurses, or graduate students with rich clinical experience and nursing knowledge working together with doctors and nurses, to achieve the integration of treatment, assessment, and education during medical treatment. Additionally, social media platforms such as WeChat in China can be used to set up nursing groups for patients with PD in China, to provide health consultation and nursing guidance. It is necessary to change patients' understanding of PD and dysphagia, help them gain correct knowledge and confidence to overcome the disease, and improve their compliance with medication and regular reexamination. It is also important that patients actively participate in multiple rehabilitation activities to delay the occurrence and progress of serious complications such as dysphagia, reduce the hospitalization rate, and improve their quality of life.

### 4.2. Paying Attention to Psychological Counseling

In this study, most patients showed obvious emotional disturbances, such as anxiety and depression, and hoped to receive more attention from their families and medical staff.

First, the disease factors of PD itself lead to poor mental health conditions, such as anxiety and depression. Second, the side effects of PD medication tend to aggravate anxiety and depression. In addition, the complexity of the treatment process during the pandemic caused anxiety and depression. In the present study, some relatives of elderly patients mistakenly believed that the patients' anxiety and depression were under their control, and they attributed these to the patients' delusions and lack of family care and support for patients.

The disease course of PD is long, and it takes time for the drugs to take effect, and some medicines seem to have no obvious effect. Patients need more understanding and support from their families. Patients who were already suffering from a sense of powerlessness in this study were in a negative state of mind, which may easily aggravate their depression.

During hospital admission, it is necessary to educate family members about basic disease knowledge, encourage patients to receive psychological treatment, and provide the patients more companionship and patience. It is necessary to analyze the psychological state of the patients by integrating facial expressions, mood, and language during the interview, fully gain the trust of the patients through empathy, encourage them to adjust their emotional state through multiple channels, give play to their subjective initiative, and help them gain a sense of self-respect in the process of emotional self-management.

We also found that the two younger interviewees had mild symptoms, but they expressed obvious concern about their self-image, and even more patients expressed anxiety. However, they were aware of their poor mental health and began to pay attention to self-regulation, which was not related to their educational background. After the interview, many respondents expressed their affirmation of the interview experience and hoped that they would have more opportunities to discuss and talk about their emotional reactions to the disease and be guided in their coping methods in the future. Some studies have applied singing therapy [32–37] and dance therapy [34] to improve swallowing, movement, and other functions in PD patients, in addition to providing the patients a pleasant experience, psychological benefit, and improving their confidence and quality of life.

### 4.3. A Program for Early Prevention of OD Complications

Standardized screening of dysphagia and early intervention are the focus of PD treatment. As early as in the Huangdi Neijing, an ancient Chinese classic, the thought of “preventive treatment of disease” was put forward. The word “prevention” highlighted in the implementation of the Medium- and Long-term Plan for the Prevention and Treatment of Chronic Diseases in China and the Healthy China Initiative 2019–2030 is consistent with the theory of “preventive treatment of diseases” in traditional Chinese medicine. Therefore, it is critical to advocate early evaluation, early intervention, and treatment of the disease before onset.

Kurpershoek et al. [38] conducted in-depth interviews with 20 patients with PD to understand their needs and wishes for an early nursing intervention plan. The results revealed that most people realized that their neurologists mainly focused on drug treatment and had little time to solve their needs for more comprehensive methods of living with Parkinson's disease, indicating that they lacked supportive nursing guidance. The patients hoped to discuss the early intervention nursing plan with medical staff in the early stage of the disease, so they could better understand the uncertainties they would face in the future.

The results of the present study show that most patients have a positive need for early intervention for PD complicated with dysphagia. For some patients, this was because of their lack of understanding, followed by the impact of the family support system. In the interview process, one respondent was elderly but had strong disease prevention awareness. The respondent had participated in group singing activities to exercise the swallowing muscles, which was related to the high cultural level and correct disease awareness of the respondent. Kurpershoek et al. [38] proved that patients can improve their swallowing and their severity of excessive salivation through routine swallowing training combined with voice training; however, the importance of early intervention was not emphasized in the study. Due to the early occult dysphagia in patients with PD, the intervention time is too late when there is obvious dysphagia. In 2017, Stegemoller et al. [34] applied singing therapy to patients with PD without obvious dysphagia for the first time. The results revealed that group singing behavior could prolong the time of laryngeal elevation and improve the emotional symptoms of patients with PD, proving that it was a good early intervention strategy.

In December 2021, the State Council issued the 14th Five-Year Plan for the Development of the National Aging Career Development and Elderly Care Service System, [39] which put forward specific requirements to improve health education and the health literacy of the elderly and strengthened the early screening, intervention, and classification guidance of key chronic diseases in the elderly. At present, researchers in China have not paid sufficient attention to the early intervention of PD, but they can use the principle of singing therapy to develop personalized early intervention programs for patients with PD with common occult dysphagia in the context of the pandemic, based on the characteristics of Chinese people. While improving disease awareness among the respondents, the interviews conducted during this study helped prevent or slow down the occurrence of complications, such as dysphagia, improved anxiety, depression, and other harmful emotions of patients and improved their quality of life.

## 5. Conclusion

At present, there are few national and international qualitative studies on PD, especially regarding the awareness of disease complications among patients. In the present study, we used descriptive phenomenological methods to conduct in-depth interviews with patients with PD to understand their cognitive status of dysphagia complications and the need for early intervention. The results of the present study show that most patients lack correct awareness of PD symptoms and early dysphagia, but they show a high need for early intervention. When patients with PD suffer from severe dysphagia, they may have severe pneumonia and face repeated hospitalization and difficulty in recovery, which may lead to economic and mental pressure on the family and society. Hospitals need to set up outpatient nurses for PD as soon as possible to provide health guidance and assessment for patients and provide resources and ways for further early intervention measures.

A limitation of this study is that all the respondents were only patients with PD from among the outpatients, and in case of some of the patients, their medication was handled by their family members, but they were not interviewed. These limitations will be taken into account in future studies.

## Figures and Tables

**Table 1:** Basic information of the interviewee.

Code	Gender	Age	Occupation	Educational background	Payment method	Course of disease	Staging of H-Y	UPDRS part II salivation score	Types of anti-Parkinsondrugs
N1	Female	77	Retired	University	Medical insurance	2.5	1	1	1.2.3
N2	Female	43	Supermarket clerk	Middle school	Medical insurance	2	2	1	1.2.3
N3	Female	56	Retired	Middle school	Medical insurance	2	1	1	1.2.3
N4	Male	45	Agency	University	Medical insurance	1	1	1	1.3
N5	Male	52	Driver	Middle school	Medical insurance	2	1	1	1.2.3
N6	Female	72	Farmer	Illiterate	Self-paid	10	2	2	1.2.3.5
N7	Male	75	Cashier	Primary school	Self-paid	17	2	1	1.2.3.5
N8	Male	67	Retired	Middle school	Medical insurance	4	1	3	1.3
N9	Male	76	Buddhist monk	Senior high school	Self-paid	6	2	1	1.2.3
N10	Female	74	Farmer	Primary school	Self-paid	12	1	1	1.2.3.5
N11	Male	80	Unemployed	Illiteracy	Self-paid	15	2	3	1.2.3.5
N12	Male	58	Businessman	Middle school	Medical insurance	3	1	1	1.3
N13	Male	49	Retired	Middle school	Medical insurance	1	2	1	2
N14	Female	68	Unemployed	Middle school	Self-paid	3	2	1	2
N15	Male	75	Veteran	University	Medical insurance	5	1	2	1.2.3
N16	Male	78	Unemployed	Primary school	Self-paid	8	1	1	1.2.3
N17	Female	74	Retired	Middle school	Medical insurance	6	1	1	1.2.3
N18	Male	72	Retired	Primary school	Medical insurance	8	1	1	1.2.3

*Note*. Types of anti-Parkinson drugs: 1. Anticholinergics; 2. Amantadine; 3. Dopamine; 4. DR agonist; 5. Monoamine oxidase-B inhibitors; 6. COMT inhibitors.

**Table 2:** Respondent results.

Topics	Subtopics	
Topic 1: lack of knowledge about PD and dysphagia	(1). Insufficient awareness of common symptoms of PD	
	(2). No awareness of excessive salivation, the main symptoms of oropharyngeal dysphagia	① The doctor was not informed
		② The symptoms were ignored
		③ Too much concern over personal image
	(3). The danger of oropharyngeal dysphagia is unclear	① There is no relevant guidance when visiting a doctor
		② Lacked knowledge acquisition channels
		③ Lacked awareness of review
		④ Expressed comprehension
	(4). Lack of coping measures for symptoms of dysphagia	

Topic 2: emotional cognitive changes	(1). Anxiety and depression	
	(2). Sense of powerlessness	

Topic 3: low awareness on the need for early intervention for dysphagia	(1). Interested in intervention	
	(2). Not interested in intervention	
	(3). Concerns	

## Data Availability

The datasets used and/or analyzed during the current study are available from the corresponding author on reasonable request.
